# Supplementary material for: Rapid, adaptable and sensitive Cas13-based COVID-19 diagnostics using ADESSO
Source: Nat Commun. 2022 Jun 8;13:3308. doi: 10.1038/s41467-022-30862-y (PMC9176161; doi:10.1038/s41467-022-30862-y)
Supplement: Supplementary file 1 — Supplementary Information [file 41467_2022_30862_MOESM1_ESM.pdf]

SUPPLEMENTARY INFORMATION

Rapid, adaptable and sensitive Cas13-based COVID-19 diagnostics using ADESSO

Casati *et al.*

Table of content:

1. Supplementary Figures pages 2-11
2. Supplementary Tables pages 12-33
3. Supplementary Methods pages 34-40
4. Supplementary References page 41

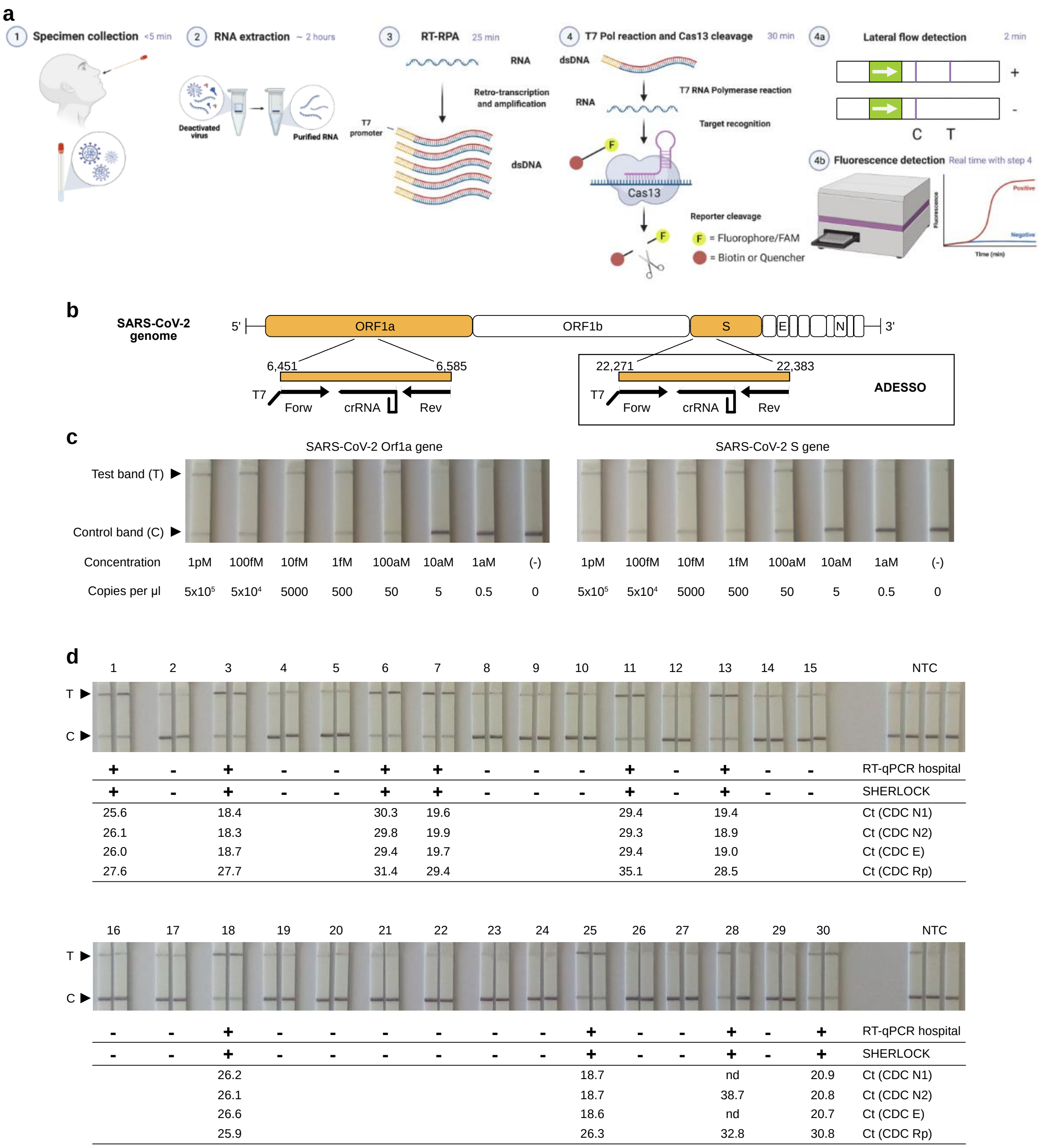

Supplementary Fig. 1: First evaluation of SHERLOCK on clinical samples. a. Graphic of SHERLOCK experimental workflow with lateral flow readout to detect SARS-CoV-2 in RNA extracted from clinical samples. **b**. Schematic of the SARS-CoV-2 genome and SHERLOCK assay locations. The regions targeted in panel **c** are highlighted in orange. Black arrows represent primers and crRNAs. Primers and crRNA targeting S were also used for ADESSO in the experiments shown in Figures 1, 2 and 3. T7 = T7 polymerase promoter; Forw = forward primer; Rev = reverse primer. **c**. SHERLOCK sensitivity on serial dilutions of an IVT fragment of SARS-CoV-2 S and Orf1a genes. **d.** Comparison of SARS-CoV-2 detection on RNA extracted from 30 clinical samples via SHERLOCK and RT-qPCR (Medical University Hospital Mannheim (RT-

qPCR hospital) or CDC 2019-nCoV Real-Time RT-PCR Diagnostic Panel). SHERLOCK was performed on SARS-CoV-2 S gene; RT-qPCR at the Medical University Hospital Mannheim was performed on SARS-CoV-2 E and Orf1a genes; CDC RT-qPCR was performed on SARS-CoV-2 N1, N2 and E genes (CDC N1, N2, E) and human RNase P (CDC Rp) as RNA quality control. T = test band; C = control band; nd = not detected; NTC = non template control.

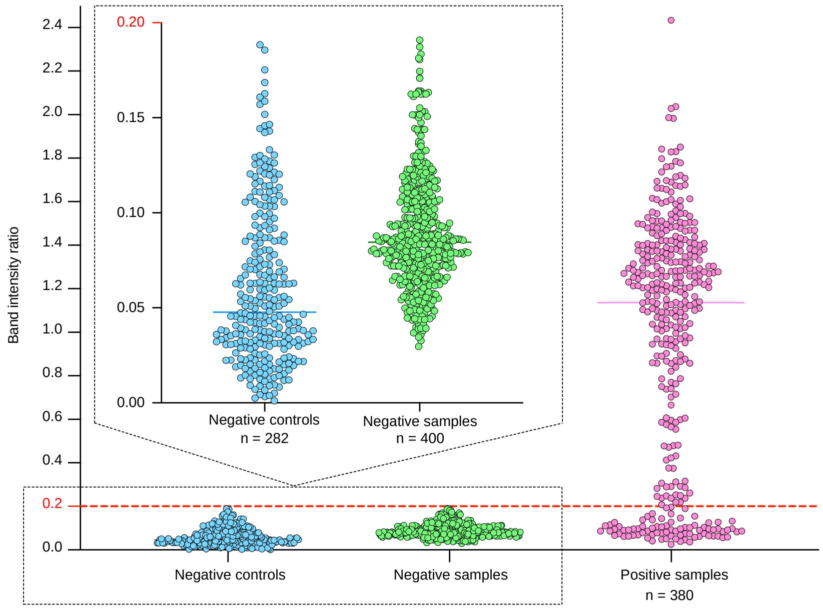

Supplementary Fig. 2: SARS-CoV-2 clinical samples. Definition of threshold between positive and negative results. The bar plot shows the band intensity ratios of all the negative controls utilized in this study (282, in blue) together with the negative (100 samples x 2 sampling methods (swab and gargle) x 2 tests (direct ADESSO and ADESSO on RNA) = 400, in green) and positive (95 samples x 2 sampling methods (swab and gargle) x 2 tests (direct ADESSO and ADESSO on RNA) = 380, in pink) clinical samples analyzed in Fig. 2. These data were used to define a threshold band intensity ratio of 0.2 to distinguish between positive and negative samples. The horizontal lines represent the median of the band intensity ratios among all the samples belonging to each category.

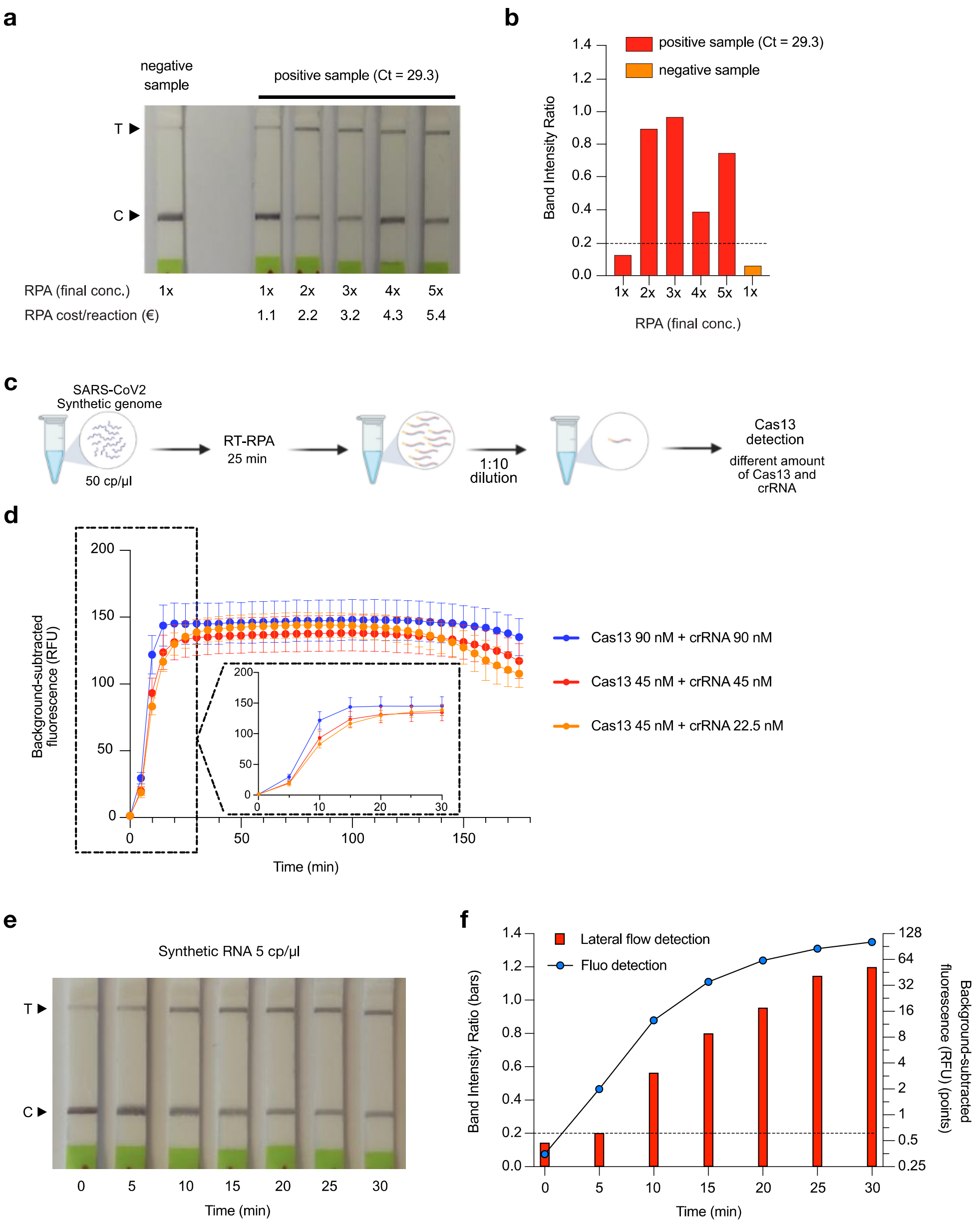

Supplementary Fig. 3: ADESSO: an optimized and highly sensitive SHERLOCK assay. a. Optimization of direct SHERLOCK sensitivity with lateral flow readout by increasing the RPA reagents to detect a false negative sample (Sample #11 in Supplementary Fig. 1, Supplementary Table 1). A true negative sample (Sample #10 in Supplementary Fig. 1, Supplementary Table 1) is used as negative control. The RPA cost per reaction for different RPA concentrations is also indicated (rounded values). For the precise values see Supplementary Table 5. **b**. Intensity ratios of the lateral flow strips in **a** are shown as a bar plot. **c**. Scheme of the experiment for the optimization of the Cas13 reaction kinetics with fluorescence readout using different amounts of Cas13 protein and crRNA. **d**. 3-hour analysis of the fluorescence signal at different time-points of the experiment depicted in **c**. The enlarged window shows the first 30 minutes of the reaction. **e**. Time-point analysis of the Cas13 reaction to determine the shortest incubation time required to detect a positive signal with lateral flow readout. **f**. Intensity ratios of the lateral flow strips in **e** are

shown in a bar plot. For panels **a** and **e**: T = test band; C = control band. For panel **d**: the bullets represent the mean of 4 technical replicates and the error bars represent the standard deviation. For panel **f**: the bullets represent the mean of 4 technical replicates.

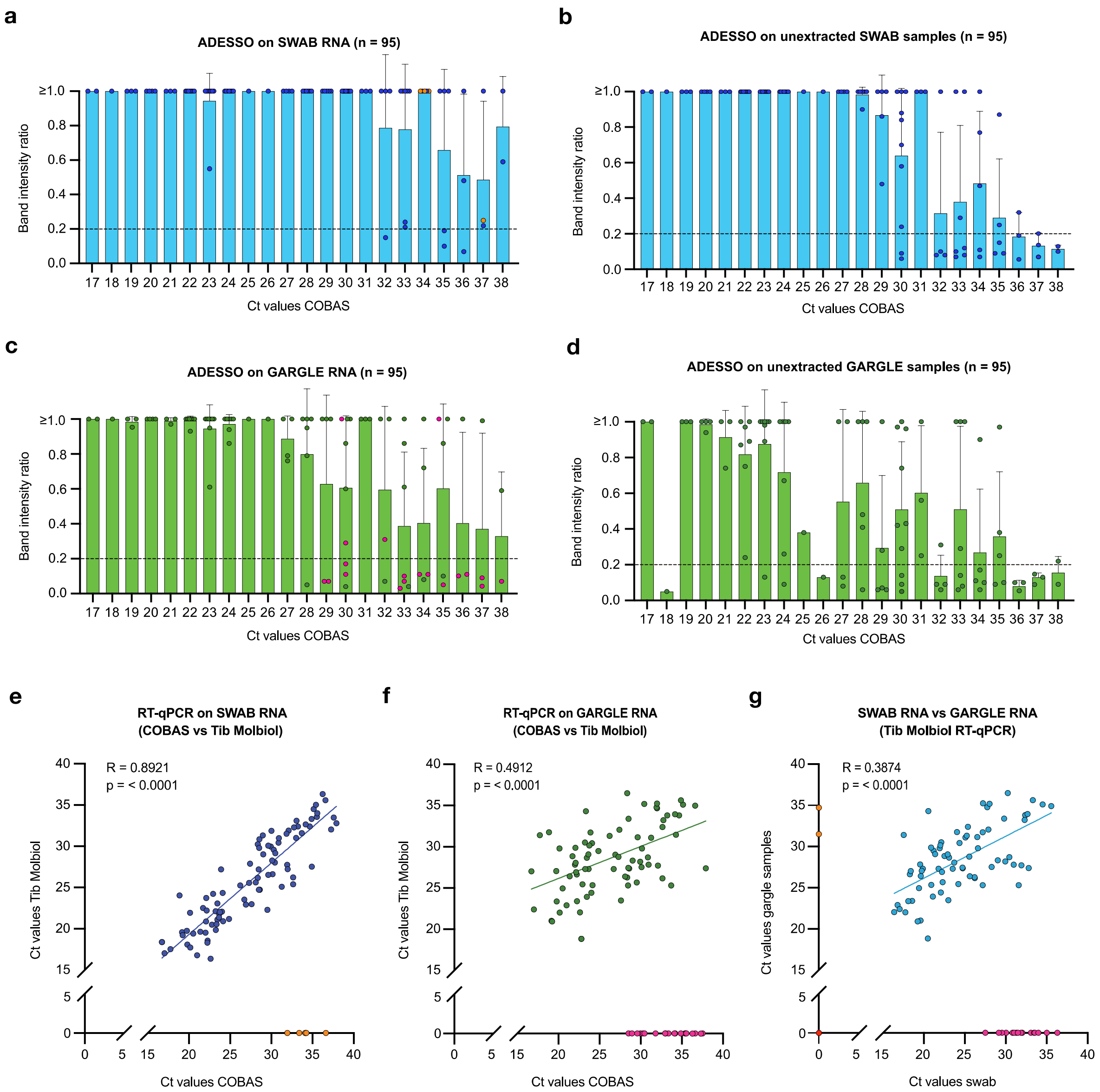

Supplementary Fig. 4: Evaluation of ADESSO performance on clinical samples in direct comparison to RT-qPCR. a. ADESSO performance on RNA extracted from swab specimens in comparison with COBAS RT-qPCR. Negative samples by Tib Molbiol RT-qPCR are represented in orange. **b**. ADESSO performance on unextracted swab specimens in comparison with COBAS RT-qPCR (performed on RNA extracted from swabs). **c**. ADESSO performance on RNA extracted from gargle samples in comparison with COBAS RT-qPCR (performed on RNA extracted from swabs). Negative samples by Tib Molbiol RT-qPCR are represented in pink. **d**. ADESSO performance on unextracted gargle samples in comparison with COBAS RT-qPCR (performed on RNA extracted from swabs). **e**. Pearson correlation (R) analysis for Ct values obtained with Tib Molbiol RT-qPCR (y axis) or COBAS RT-qPCR (x axis) on RNA extracted from swab specimens. Negative samples by Tib Molbiol RT-qPCR are represented in orange and are excluded in the calculation of the correlation (R). **f**. Pearson correlation analysis of Ct values obtained with Tib Molbiol RT-qPCR (y axis) and COBAS RT-qPCR (x axis) on RNA extracted

from gargle samples. Negative samples by Tib Molbiol RT-qPCR are represented in pink and are excluded in the calculation of the correlation (R). **g**. Pearson correlation analysis of Ct values obtained after Tib Molbiol RT-qPCR on RNA extracted from gargle (y axis) and swab (x axis) samples. Negative swab samples are represented in orange; negative gargle samples are represented in pink; negative samples both as gargle and swab are represented in red. All the negative samples are excluded from the calculation of the correlation (R). For all the Pearson correlation (R) analysis a two-tailed p-value was calculated. In panels **a**-**d** only the band intensity ratios of the positive samples are shown (n = 95). Values higher than 1 are plotted as equal to 1 for better visualization. Ct values are rounded to the nearest whole number for the sake of visualization. The exact Ct values are listed in Supplementary Table 2. For panels **a**, **b**, **c** and **d**: the bars represent the mean Ct value among all the clinical samples belonging to each category of Ct value and the error bars represent the positive standard deviation.

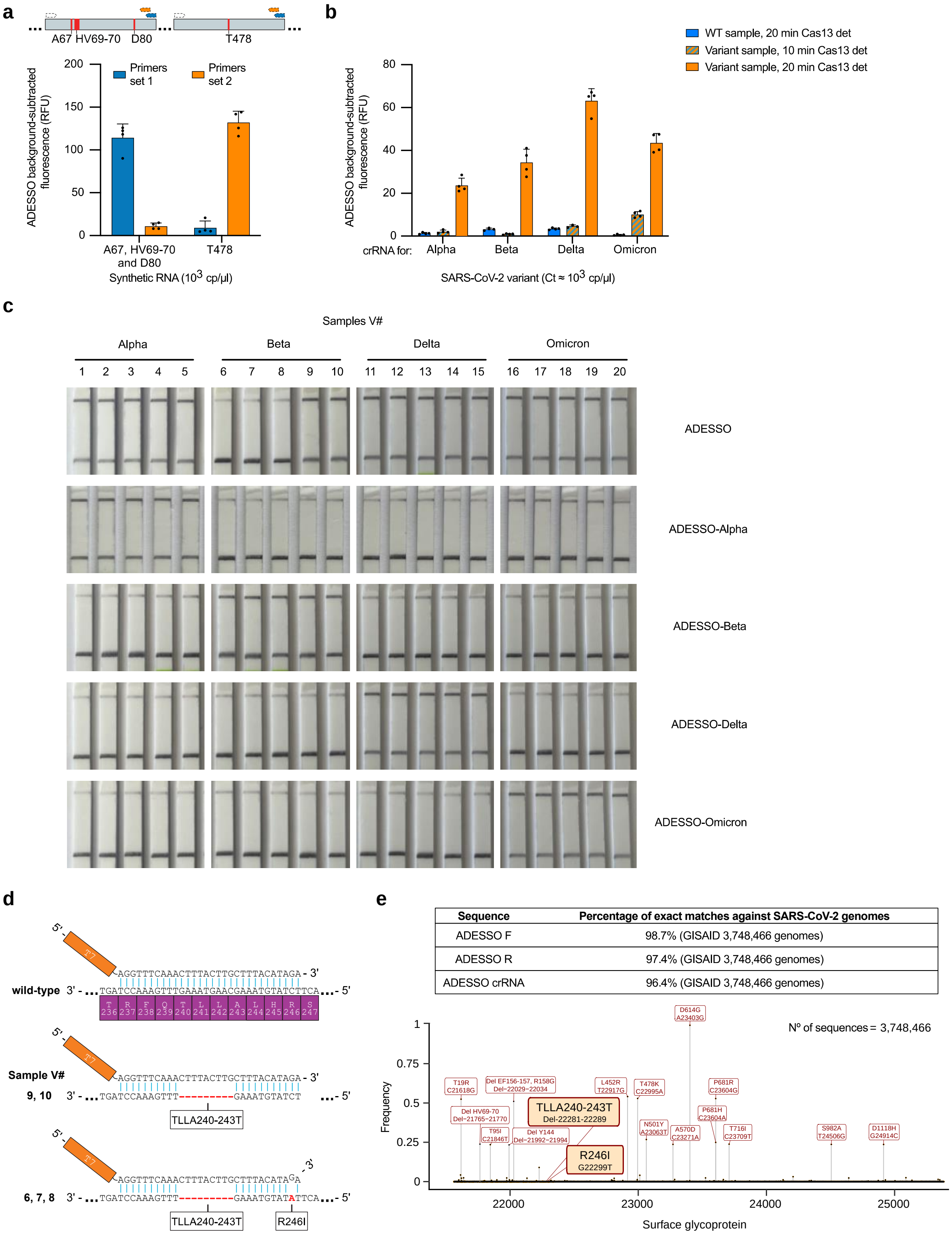

Supplementary Fig. 5: Adaptation of ADESSO for detection of SARS-CoV-2 variants. a. RT-RPA primers optimization to amplify the region of SARS-CoV-2 S gene containing mutations at A67, HV69-70 and D80 or T478. Two combinations of the same forward primer (white arrow) with two alternative reverse primers were tested (reverse 1 in blue and reverse 2 in orange) on a 10^3

cp/µl dilution of SARS-CoV-2 synthetic genome (wild-type sequence). Cas13 detection was performed using crRNAs specific for HV69-70 and T478. Bars represent the mean of 4 technical replicates and the error bars represent the standard deviation. **b**. ADESSO fluorescence using specific crRNAs for the detection of different variants. Clinical samples carrying the WT virus or specific variant with a Ct value corresponding to 10^3 cp/µl were used as targets. **c**. Variant specific ADESSO tests on 20 clinical samples carrying different variants. The band intensity ratios of the sticks depicted here are shown in Fig. 3b. **d**. Schematic of the binding of the forward RPA primer used in ADESSO to the complementary region in the original SARS-CoV-2 sequence (top) and in Beta samples V#6-10. Samples V#9-10 carry the deletion Δ242-244 only (middle), while samples V#6-8 have an additional mutation (R246I) (bottom). The positions of the Δ242-244 deletion and the R246I mutation are highlighted in gray. The mutations causing amino acid changes are marked in red. **e**. Bioinformatic analysis showing the percentage of exact target matches for RT-RPA primers and crRNA used for ADESSO (top) and the mutation frequency for each position in the S gene (bottom). Mutations leading to Δ242-244 and R246I are highlighted. For panels **a** and **b**: bars represent the mean of n = 4 technical replicates and error bars represent the positive standard deviation.

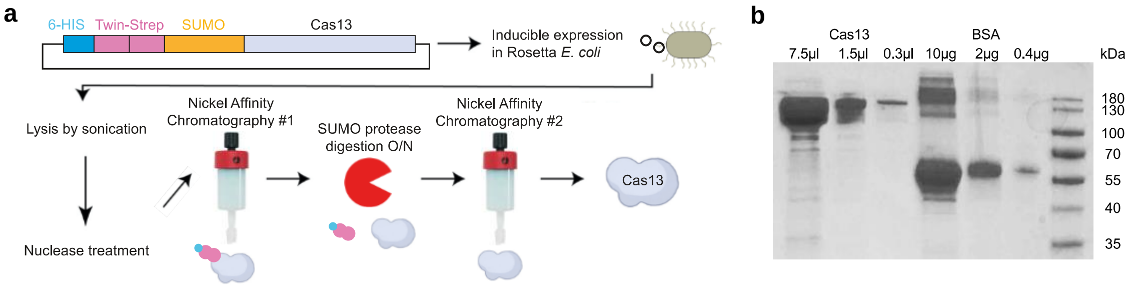

Supplementary Fig. 6: Generation of LwaCas13a. a. LwaCas13a protein purification. The LwaCas13 fusion construct also encodes multiple affinity tags and a protease recognition site at the N terminus of the polypeptide. We have utilized the 6xHIS tag as the basis for our relatively inexpensive purification, while others have developed an alternative protocol based on the Strep-tags[1]. After expression in Rosetta cells (inducible via the Lac operon), the cells are lysed by sonication and the nucleic acid contained within the lysate is digested. The fusion protein is then purified by nickel-affinity chromatography. The purified fusion protein is digested with SUMO protease, which cleaves the tags and majority of the SUMO sites off of the mature protein. The SUMO protease and in-tact affinity tags are then removed from the sample by re-applying the sample to the nickel column, leaving >98% pure Cas13. We also employ a size exclusion chromatography step to remove any aggregated Cas13 protein (not pictured). **b**. Serially diluted amounts of pure Cas13 were analyzed by coomassie staining after conventional SDS-PAGE, revealing a prominent band at the appropriate molecular weight and only minor contaminants. A serial dilution of BSA was also run as an estimate of protein concentration by densitometry (which was also validated by BCA assay). Two different high-yielding purifications were used to generate the results present throughout this study, both of which yielded proteins that had comparable levels of activity.

Sample ID	CDC N1 (Ct)	CDC N2 (Ct)	CDC E (Ct)	CDC Rp (Ct)	SHERLOCK	Notes
1	25.6	26.1	26	27.6	+	
2	nd	nd	nd	nd	-	
3	18.4	18.3	18.7	27.7	+	
4	nd	nd	nd	nd	-	
5	nd	nd	nd	nd	-	
6	30.3	29.8	29.4	31.4	+	
7	19.6	19.9	19.7	29.4	+	
8	nd	nd	nd	nd	-	
9	nd	nd	nd	nd	-	
10	nd	nd	nd	nd	-	Sample used also in S3a
11	29.4	29.3	29.4	35.1	+	Sample used also in S3a
12	nd	nd	nd	nd	-	
13	19.4	18.9	19	28.5	+	
14	nd	nd	nd	nd	-	
15	nd	nd	nd	nd	-	
16	nd	nd	nd	nd	-	
17	nd	nd	nd	nd	-	
18	26.2	26.1	26.6	25.9	+	
19	nd	nd	nd	nd	-	
20	nd	nd	nd	nd	-	
21	nd	nd	nd	nd	-	
22	nd	nd	nd	nd	-	
23	nd	nd	nd	nd	-	
24	nd	nd	nd	nd	-	
25	18.7	18.7	18.6	26.3	+	

26	nd	nd	nd	nd	-	
27	nd	nd	nd	nd	-	
28	nd	38.7	nd	32.8	+	
29	nd	nd	nd	nd	-	
30	20.9	20.8	20.7	30.8	+	

Supplementary Table 1: Clinical information of samples used in Supplementary Fig. 1.
+ = positive test; - = negative test; nd = not detected.

	SWAB					GARGLE WATER			
	RNA			Lysate		RNA		Lysate	
Sample ID	COBAS (Ct)	Tib MolBiol (Ct)	ADESSO	direct ADESSO	Antigen test	Tib MolBiol (Ct)	ADESSO	direct ADESSO	Antigen test
316	35.15	34.5	1.53	0.09	-	35.1	0.10	0.10	-
327	21.72	22.53	1.59	1.69	+	28.85	1.27	0.24	-
329	23.46	23.07	1.42	2.03	+	28.66	1.20	1.47	-
339	31.98	nd	0.82	0.07	-	34.68	0.60	0.11	-
345	28.55	30.84	2.43	1.09	-	nd	0.07	0.06	-
359	29.93	29.96	1.41	0.84	-	28.18	0.99	0.96	-
363	28.62	24.74	1.61	0.86	+	27.37	1.40	0.28	-
376	22.63	16.38	1.25	1.84	+	22.04	0.95	1.11	-
409	28.49	24.73	2.04	1.30	-	27.44	0.95	1.13	-
414	22.28	18.57	1.98	1.76	+	23.39	0.93	0.89	-
417	22.03	19.54	1.37	1.67	+	27.84	1.02	0.87	-
436	34.9	33.32	0.10	0.09	-	35.63	0.86	0.25	-
437	27.61	23	1.62	1.67	+	23.49	0.79	1.50	-
461	32.64	26.23	1.41	0.29	-	26.17	0.86	1.19	-
463	35.42	35.04	1.48	0.15	-	nd	0.05	0.38	-
464	31.92	27.74	1.66	0.08	-	34.78	0.07	0.31	-
470	37.35	32.03	0.22	0.20	-	nd	0.04	0.15	-
530	33.07	33.08	0.21	0.07	-	nd	0.03	1.02	-
535	30.43	28.02	1.53	1.54	-	35.2	0.04	0.05	-
536	28.97	31.31	1.38	1.38	-	nd	0.07	0.06	-
539	24.09	24.35	1.64	1.51	-	30.43	0.86	0.67	-
546	33.22	32.4	1.61	0.12	-	33.82	0.04	0.06	-
549	28.35	30.24	1.66	1.71	-	36.49	0.05	0.06	-
564	34.66	32.39	0.19	0.25	-	33.82	1.26	0.97	-

602	32.1	31.91	0.15	1.28	-	27.71	1.09	0.09	-
622	28.47	27.2	1.85	1.36	+	25.32	1.23	1.43	-
624	36.64	35.56	1.11	0.14	-	34.93	0.97	0.09	-
625	28.36	26.18	1.38	1.55	-	26.28	1.23	0.41	-
661	23.45	21.1	1.59	1.71	+	23.93	1.28	1.83	-
666	34.18	32.66	1.30	0.47	-	33.95	0.72	0.17	-
677	19.8	18.1	1.83	1.46	+	23.38	1.33	1.58	-
679	37.57	33.47	1.30	0.13	-	nd	0.07	0.09	-
703	23.23	22.14	1.72	1.80	+	30.53	1.01	0.13	-
704	35.52	33.56	0.48	0.19	-	nd	0.11	0.10	-
717	26.92	22.92	1.51	1.61	+	29.06	0.79	1.74	-
727	23.69	21.81	1.69	1.99	+	27.31	1.37	0.26	-
734	33.71	31.59	1.44	0.77	-	25.32	0.08	0.90	-
735	24.14	20.9	1.70	1.19	+	25.33	1.15	1.28	-
738	22.8	20.5	1.55	1.10	+	18.84	1.02	0.98	+
740	22.25	18.3	1.66	1.09	+	26.65	1.02	0.97	-
741	20.53	19.41	1.53	1.09	+	24.94	1.04	0.74	-
748	30.01	31.88	1.43	0.70	-	nd	0.17	0.09	-
757	30.1	28.28	1.51	0.58	+	27.52	0.86	0.97	-
769	31.99	27.22	1.47	0.08	-	35.26	1.25	0.06	-
812	28.1	25.62	1.21	0.90	+	26.39	1.07	0.48	-
814	30.17	26.62	1.13	0.88	-	29.81	0.60	0.43	-
835	27.3	25.55	1.14	1.48	+	28.77	0.76	0.13	-
845	37.91	32.8	0.59	0.10	-	27.4	0.59	0.22	-
850	24.86	21.2	1.27	1.68	+	29.48	1.51	0.38	-
859	34.93	27.52	1.23	0.87	-	nd	1.48	0.09	-
902	32.64	25.4	1.40	1.32	-	32.39	0.61	0.29	-

904	23.36	20.56	1.41	1.79	+	34.32	0.61	0.89	-
909	29.53	22.29	1.40	1.41	+	25.68	1.61	1.27	-
2001	33.42	nd	1.29	0.08	-	nd	0.10	0.08	-
2004	19.18	19.59	1.26	1.55	+	21.03	1.14	1.32	+
2007	17.76	17.54	1.41	1.51	+	31.41	1.28	0.05	-
2023	31.26	25.09	1.28	1.54	-	31.11	1.35	0.25	-
2037	22.08	20.88	1.50	1.78	+	29.88	1.25	0.75	-
2048	26.72	23.53	1.07	1.38	+	27.32	1.35	1.04	-
2057	24	22.08	1.33	1.37	+	24.44	1.76	1.10	-
2059	34.24	nd	1.27	0.07	-	nd	0.11	0.06	-
2064	20.99	16.78	1.32	1.37	+	22.9	1.13	1.13	-
2068	20.13	21.93	1.27	1.22	+	29.8	1.33	0.94	-
2071	20.17	17.74	1.26	1.48	+	22.02	1.07	1.50	+
3000	28.53	29.81	1.37	0.48	-	33.41	1.17	0.07	-
3005	17.04	17.04	1.50	1.48	+	22.41	1.04	1.32	+
3009	26.24	25.26	1.28	1.17	+	31.31	1.09	0.13	-
3010	16.72	18.4	1.22	1.26	+	27.03	1.05	1.34	-
3011	23.75	22.02	1.36	1.40	+	30.15	1.12	1.29	-
3013	23.26	19.87	1.30	1.47	+	27.32	1.06	1.14	-
3019	21.38	19.64	1.56	1.36	+	26.41	0.97	1.22	-
3027	33	31.02	0.24	1.50	-	nd	0.07	0.14	-
4000	23.66	21.37	1.10	1.44	+	29.09	0.94	1.26	-
G2	36.32	36.32	0.06	0.06	-	nd	0.10	0.05	-
G3	30.97	30.97	1.20	1.26	-	27.8	1.20	1.11	-
G4	19.23	19.23	1.20	1.21	+	20.92	0.95	1.19	+
X-11	36.67	nd	0.25	0.07	-	nd	0.09	0.13	-
X-17	29.62	30.07	1.29	0.24	-	nd	0.11	0.14	-

X-18	34.15	nd	1.23	1.14	-	nd	0.11	0.11	-
X-21	30.87	30.44	1.21	1.22	-	28.71	1.32	0.56	-
X-22	22.94	24.23	0.55	1.13	-	25.45	1.45	1.19	-
X-25	29.62	26.53	1.40	1.03	-	31.15	1.28	0.74	-
X-28	22.17	22.21	1.27	1.37	+	26.93	1.07	1.35	-
X-33	27.41	27.89	1.38	1.44	-	32.08	1.38	0.08	-
X-34	29.02	29.03	1.34	1.12	-	28.34	1.20	1.21	-
X-53	18.86	24.04	1.16	1.49	+	31.75	1.26	1.17	-
X-55	19.76	19.75	1.28	1.60	+	27.05	1.14	1.25	-
X-58	32.76	32.3	1.10	0.10	-	33.23	1.32	1.02	-
X-66	30.31	29.57	1.48	0.09	+	nd	1.23	0.42	-
X-72	30.5	29.17	1.40	0.06	-	nd	0.29	0.29	-
X-78	35.59	34.01	1.51	0.32	-	nd	1.35	0.10	-
X-81	31.85	31.51	1.46	0.10	-	nd	0.31	0.09	-
X-83	22.11	23.73	1.33	1.21	+	28.04	1.39	1.21	-
X-84	34.29	nd	1.42	0.11	-	31.46	1.23	0.10	-
X-89	23.91	27.19	1.42	1.29	-	31.73	1.31	0.09	-

Supplementary Table 2: Clinical information of positive samples used in Fig. 2 and Supplementary Fig. 4.
+ = positive test; - = negative test; nd = not detected.

	SWAB					GARGLE WATER				
	RNA			Lysate		RNA		Lysate		
Sample ID	COBAS (Ct)	Tib MolBiol (Ct)	ADESSO	direct ADESSO	Antigen test	Tib MolBiol (Ct)	ADESSO	direct ADESSO	Antigen test	Notes
300	nd	nd	0.07	0.05	-	nd	0.16	0.10	-	Used also in Figure 1a
301	nd	nd	0.06	0.07	-	nd	0.09	0.09	-	
302	nd	nd	0.14	0.10	-	nd	0.08	0.06	-	
303	nd	nd	0.06	0.07	-	nd	0.11	0.08	-	
304	nd	nd	0.08	0.06	-	nd	0.11	0.12	-	
305	nd	nd	0.07	0.05	-	nd	0.09	0.08	-	
307	nd	nd	0.03	0.08	-	nd	0.06	0.09	-	
308	nd	nd	0.07	0.06	-	nd	0.09	0.05	-	
309	nd	nd	0.06	0.06	-	nd	0.08	0.12	-	
310	nd	nd	0.05	0.08	-	nd	0.08	0.11	-	
311	nd	nd	0.10	0.09	-	nd	0.11	0.11	-	
312	nd	nd	0.07	0.04	-	nd	0.08	0.09	-	
313	nd	nd	0.07	0.07	-	nd	0.07	0.09	-	
314	nd	nd	0.09	0.09	-	nd	0.11	0.08	-	
315	nd	nd	0.03	0.05	-	nd	0.18	0.10	-	
317	nd	nd	0.07	0.12	-	nd	0.17	0.09	-	
318	nd	nd	0.05	0.05	-	nd	0.14	0.10	-	
319	nd	nd	0.11	0.08	-	nd	0.12	0.09	-	
320	nd	nd	0.07	0.09	-	nd	0.12	0.11	-	
321	nd	nd	0.07	0.07	-	nd	0.10	0.09	-	
322	nd	nd	0.09	0.09	-	nd	0.09	0.08	-	
323	nd	nd	0.08	0.11	-	nd	0.16	0.11	-	
324	nd	nd	0.07	0.08	-	nd	0.08	0.13	-	

325	nd	nd	0.04	0.11	-	nd	0.11	0.12	-	
326	nd	nd	0.05	0.10	-	nd	0.11	0.12	-	
328	nd	nd	0.06	0.09	-	nd	0.11	0.09	-	
330	nd	nd	0.08	0.08	-	nd	0.08	0.09	-	
331	nd	nd	0.08	0.08	-	nd	0.08	0.05	-	
333	nd	nd	0.08	0.09	-	nd	0.08	0.10	-	
335	nd	nd	0.07	0.05	-	nd	0.09	0.08	-	
336	nd	nd	0.04	0.06	-	nd	0.09	0.15	-	
337	nd	nd	0.08	0.04	-	nd	0.06	0.08	-	
338	nd	nd	0.12	0.14	-	nd	0.04	0.07	-	
340	nd	nd	0.09	0.06	-	nd	0.05	0.09	-	
341	nd	nd	0.09	0.06	-	nd	0.05	0.08	-	
342	nd	nd	0.07	0.05	-	nd	0.07	0.11	-	
343	nd	nd	0.13	0.08	-	nd	0.07	0.08	-	
346	nd	nd	0.08	0.08	-	nd	0.07	0.04	-	
347	nd	nd	0.13	0.06	-	nd	0.07	0.08	-	
348	nd	nd	0.08	0.07	-	nd	0.05	0.08	-	
350	nd	nd	0.15	0.05	-	nd	0.07	0.08	-	
351	nd	nd	0.11	0.09	-	nd	0.07	0.08	-	
352	nd	nd	0.10	0.07	-	nd	0.04	0.06	-	
353	nd	nd	0.06	0.06	-	nd	0.04	0.07	-	
354	nd	nd	0.09	0.11	-	nd	0.07	0.07	-	
355	nd	nd	0.12	0.09	-	nd	0.03	0.06	-	
356	nd	nd	0.12	0.06	-	nd	0.05	0.08	-	
358	nd	nd	0.12	0.04	-	nd	0.09	0.09	-	
360	nd	nd	0.07	0.07	-	nd	0.06	0.09	-	
362	nd	nd	0.13	0.05	-	nd	0.05	0.09	-	

364	nd	nd	0.12	0.06	-	nd	0.05	0.07	-	
365	nd	nd	0.09	0.05	-	nd	0.04	0.07	-	
366	nd	nd	0.06	0.07	-	nd	0.05	0.06	-	
367	nd	nd	0.08	0.10	-	nd	0.04	0.08	-	
368	nd	nd	0.11	0.06	-	nd	0.08	0.08	-	
369	nd	nd	0.07	0.05	-	nd	0.08	0.04	-	
370	nd	nd	0.15	0.04	-	nd	0.15	0.06	-	
371	nd	nd	0.15	0.05	-	nd	0.08	0.07	-	
372	nd	nd	0.05	0.08	-	nd	0.12	0.09	-	
373	nd	nd	0.10	0.05	-	nd	0.06	0.10	-	
374	nd	nd	0.07	0.10	-	nd	0.19	0.06	-	
375	nd	nd	0.10	0.11	-	nd	0.11	0.08	-	
377	nd	nd	0.08	0.10	-	nd	0.12	0.09	-	
378	nd	nd	0.12	0.12	-	nd	0.11	0.06	-	
379	nd	nd	0.06	0.11	-	nd	0.16	0.08	-	
380	nd	nd	0.11	0.09	-	nd	0.16	0.08	-	
381	nd	nd	0.12	0.11	-	nd	0.10	0.09	-	
382	nd	nd	0.10	0.12	-	nd	0.13	0.14	-	
383	nd	nd	0.15	0.12	-	nd	0.16	0.11	-	
384	nd	nd	0.16	0.08	-	nd	0.11	0.10	-	
385	nd	nd	0.10	0.12	+	nd	0.13	0.12	-	
400	nd	nd	0.07	0.08	-	nd	0.09	0.07	-	
402	nd	nd	0.12	0.12	-	nd	0.13	0.12	-	
403	nd	nd	0.10	0.14	-	nd	0.15	0.11	-	
404	nd	nd	0.09	0.09	-	nd	0.08	0.10	-	
405	nd	nd	0.10	0.14	-	nd	0.08	0.07	-	
406	nd	nd	0.11	0.10	-	nd	0.08	0.13	-	

407	nd	nd	0.12	0.08	-	nd	0.09	0.10	-	
408	nd	nd	0.09	0.12	-	nd	0.09	0.09	-	
410	nd	nd	0.08	0.08	-	nd	0.11	0.12	-	
411	nd	nd	0.09	0.09	-	nd	0.09	0.07	-	
412	nd	nd	0.11	0.08	-	nd	0.07	0.08	-	
413	nd	nd	0.09	0.08	-	nd	0.11	0.04	-	
415	nd	nd	0.14	0.08	-	nd	0.16	0.06	-	
416	nd	nd	0.15	0.08	-	nd	0.16	0.07	-	
418	nd	nd	0.16	0.09	-	nd	0.08	0.08	-	
420	nd	nd	0.06	0.08	-	nd	0.12	0.08	-	
421	nd	nd	0.07	0.06	-	nd	0.04	0.07	-	
422	nd	nd	0.08	0.05	-	nd	0.08	0.05	-	
423	nd	nd	0.18	0.12	-	nd	0.19	0.08	-	
424	nd	nd	0.05	0.08	-	nd	0.12	0.06	-	
425	nd	nd	0.09	0.10	-	nd	0.12	0.07	-	
426	nd	nd	0.09	0.10	-	nd	0.13	0.06	-	
427	nd	nd	0.10	0.13	-	nd	0.10	0.09	-	
428	nd	nd	0.12	0.10	-	nd	0.06	0.07	-	
429	nd	nd	0.13	0.11	-	nd	0.12	0.06	-	
430	nd	nd	0.09	0.09	-	nd	0.09	0.07	-	
431	nd	nd	0.10	0.14	-	nd	0.17	0.11	-	
432	nd	nd	0.08	0.14	-	nd	0.18	0.06	-	
433	nd	nd	0.13	0.08	-	nd	0.17	0.16	-	

Supplementary Table 3: Clinical information of negative samples used in Fig. 2.
+ = positive test; - = negative test; nd = not detected.

Sample ID	Variant	COBAS (Ct)	Direct ADESSO				
			Standard	Alpha	Beta	Delta	Omicron
v1	B.1.1.7 (Alpha)	20,3	1,19	0,96	0,15	0,16	0,14
v2	B.1.1.7 (Alpha)	18,16	1,28	0,94	0,14	0,22	0,14
v3	B.1.1.7 (Alpha)	17,7	1,21	1,11	0,18	0,23	0,19
v4	B.1.1.7 (Alpha)	18,94	1,23	0,94	0,22	0,22	0,16
v5	B.1.1.7 (Alpha)	19,94	1,03	1,05	0,26	0,21	0,14
v6	B.1.351 (Beta)	15,47	0,11	0,17	0,50	0,20	0,21
v7	B.1.351 (Beta)	18,92	0,13	0,18	0,65	0,22	0,18
v8	B.1.351 (Beta)	19,42	0,19	0,19	0,77	0,16	0,17
v9	B.1.351 (Beta)	17,38	0,91	0,14	0,68	0,15	0,11
v10	B.1.351 (Beta)	17,72	0,93	0,20	0,71	0,20	0,20
v11	B.1.617.2 (Delta)	15,96	1,10	0,15	0,24	1,13	0,17
v12	B.1.617.2 (Delta)	24,18	1,02	0,17	0,15	0,97	0,24
v13	B.1.617.2 (Delta)	17,72	1,08	0,18	0,22	1,17	0,12
v14	B.1.617.2 (Delta)	20,27	1,08	0,15	0,23	1,14	0,18
v15	B.1.617.2 (Delta)	16,8	1,01	0,19	0,16	1,12	0,22
v16	B.1.1.529 (Omicron)	17,95	1,16	0,33	0,22	0,19	1,31
v17	B.1.1.529 (Omicron)	21,14	1,26	0,33	0,21	0,17	1,22
v18	B.1.1.529 (Omicron)	21,37	1,13	0,30	0,23	0,19	1,21
v19	B.1.1.529 (Omicron)	21,31	1,00	0,34	0,20	0,16	1,28
v20	B.1.1.529 (Omicron)	20,01	0,92	0,20	0,24	0,17	1,32

Supplementary Table 4: Clinical samples information of samples used in Fig. 3 and Supplementary Fig. 5. For ADESSO tests we report the band intensity ratios plotted in Fig. 3b. Tests resulting in a band intensity ratio higher than 0.4 are considered positive.

	Total cost (€)	Number of reactions	Cost per reaction (€)
**Lysis**			
QuickExtract DNA Extraction Solution (Lucigen #QE09050, 50ml)	283.1	5000	**0.06**
**RPA reaction**			
RPA pellet, Rehy. Buffer, MgOAc	405	240	1.69
F primer (5pmol per reaction; 1 tube = 20.000pmol)	6	2000	0.0030
R primer (5 pmol per reaction; 1 tube = 20.000pmol)	3	2000	0.0015
M-MuLV (200U/ul - M0253L = 50.000U; 60U per reaction)	227.2	833.33	0.27
RNase Inhibitor, Murine (40U/µl M0314L = 15.000 U; 12U per reaction)	230.4	1250	0.18
**Total cost per 1 RPA reaction**			**2.15**
**Cas13 detection (lateral flow)**			
Cleavage buffer (Sigma, #T2194-100ML, 400mM Tris pH 7.4; 1µl per reaction)	32.46	250000	0.00013
LwaCas13a protein in SB (126.6µg/ml; around 240ng per reaction)	667.25	40000	0.017
crRNA (40ng/ul)	150	32500	0.0046
Lateral-Flow-Reporter (137nmol, 20pmol per reaction) *	306.7	13700	0.02
SUPERase-In Rnase Inhibitor (10000U, 20 U/µL; 20U per reaction) *	461.36	1000	0.46
Lucigen T7 Polymerase (125,000 U; 50U/µl; # 30223-2; 29U per reaction)	578.55	8620	0.07

rNTP solution mix (25mM each) (NEB, #N0466L = 2ml) (0.8µl per reaction)	233.6	5000	0.05
$MgCl_2$ (120mM) (#63069-100ML, Sigma Aldrich) (1µl per reaction)	81	1660000	0.00005
**Total cost per 1 Cas13 lateral flow detection reaction**			**0.62**
**Cas13 detection (fluo readout)**			
Cleavage buffer (Sigma, #T2194-100ML, 400mM Tris pH 7.4; 1µl per reaction)	32.46	250000	0.00013
LwaCas13a protein in SB (126.6µg/ml; around 240ng per reaction)	667.25	40000	0.017
crRNA (40ng/ul)	150	32500	0.0046
LwaCas13a specific fluorescent reporter (from IDT, 12.9nmol) (2pmol/reaction)	328.65	6450	0.05
RNase Inhibitor, Murine (40U/µl M0314L = 15.000 U; 40U per reaction)	230.4	750	0.31
Lucigen T7 Polymerase (125,000 U; 50U/µl; # 30223-2; 29U per reaction)	578.55	8620	0.07
rNTP solution mix (25mM each) (NEB, #N0466L = 2ml) (0.8µl per reaction)	233.6	5000	0.05
$MgCl_2$ (120mM) (#63069-100ML, Sigma Aldrich) (1µl per reaction)	81	1660000	0.00005
**Total cost per 1 Cas13 fluorescence detection reaction**			**0.50**
**Milenia HybriDetect 1 (TwistDx, cat. no. MILENIA01) (only for lateral flow readout)**	205	100	**2.05**
**Total cost per 1 ADESSO reaction with lateral flow stick**			**4.88**
**Total cost per 1 ADESSO reaction with fluo readout**			**2.71**

**Cas13 purification**	**Cost of one bottle/item (€)**	**Approx. amount needed for purification**	**Approx. cost for one purification (€)**
Yeast extract	270 - 2.5 kg	125 g	13.5
Peptone	165 - 500 g	100 g	33
IPTG	230 - 5 g	600 mg	28
Ampicillin	230 - 50 g	0.5 g	2.3
Tris pH 8 1M	80 - 1 L	40 mL	3.2
Tris pH 7.5 1M	80 - 1 L	2.5 mL	0.25
NaCl	180 - 10 kg	200 g	3.4
DTT	185 - 10 g	630 mg	11
Lysozyme	215 - 10 g	600 mg	13
Benzoase	200 - 25000 units in 100 μL	6 uL	13
Sumo Protease	480 - 250 units at 1 U/μL	150 μL	288
Hepes	295 - 500 g	2.4 g	1.4
MgCl2	20 - 100 g	0.5 g	0.1
Glycerol	70 - 2.5 L	2.5 mL	0.1
Imidazole	130 - 500 g	18 g	5
HIS trap columns (high performance)	180 - pkg of 5	1	36
Protease inhibitors (EDTA free complete tablets)	360 - 20 tblts	12	216
**Total**			**667.25**

Purification yielded enough for approx. 40,000 reactions			
**Therefore total cost div by 40000 is the cost per reaction ----->**	**0.01668125**		
**Storage Buffer for Cas13a protein**	**Total cost (€)**	**Number of preps**	**Cost per prep (50mL) (€)**
Tris-HCl (pH 7.5, 1 M, 1L from Sigma Aldrich) (2.5mL per prep)	75	400	0.1875
NaCl (5 M, from 5kg in powder) (6mL per prep)	24.7	14259.63952	0.001732162
glycerol (1L) (2.5mL per prep)	19.06	400	0.04765
DTT (1M, from 25g) (100µl per prep)	118.81	1620.714022	0.073307196
UltraPure water (500mL) (38.9mL per prep)	11.81	12.85347044	0.918818
**Total cost per 1 prep (50mL --> 326.8 x 79 reactions)**			**1.229007357**
**Total cost per 1 Cas13 detection reaction**			**0.0000476**

Supplementary Table 5: ADESSO cost per reaction.

Oligos			
**Name**	**Sequence (5'->3')**	**Fig.**	**Purpose**
T7-3G oligo	gaaattaatacgactcactataggg		T7 promoter annealing oligonucleotide for IVT
S gene RT-RPA forward primer	gaaattaatacgactcactatagggAGGTTTCAAACTTTACTTGCTTTACATAGA	1-3, S1-S5	Primer for pre-amplification step using RT-RPA - S - Zhang Protocol[2]
S gene RT-RPA reverse primer	TCCTAGGTTGAAGATAACCCACATAATAAG	1-3, S1-S5	Primer for pre-amplification step using RT-RPA - S - Zhang Protocol[2]
Orf1a RT-RPA forward primer 1	gaaattaatacgactcactatagggCGAAGTTGTAGGAGACATTATACTTAAACC	S1c	Primer for pre-amplification step using RT-RPA - Orf1ab - Zhang Protocol[2]
Orf1a RT-RPA reverse primer 1	TAGTAAGACTAGAATTGTCTACATAAGCAGC	S1c	Primer for pre-amplification step using RT-RPA - Orf1ab - Zhang Protocol[2]
Orf1a gene RT-PCR forward primer	gaaattaatacgactcactatagggCGCGCAGGGAATGGATAATCTTG	S1c	Primer for producing RNA target for Orf1a
Orf1a gene RT-PCR reverse primer	GCAGCTAAACCATGAGTAGCAAGG	S1c	Primer for producing RNA target for Orf1a
S gene RT-PCR forward primer	gaaattaatacgactcactatagggATTTAGTGCGTGATCTCCCTCAGG	S1c	Primer for producing RNA target for S
S gene RT-PCR reverse primer	TTTCTGAGAGAGGGTCAAGTGCAC	S1c	Primer for producing RNA target for S
S A67, HV69-70, D80 RT-RPA forward primer	gaaattaatacgactcactatagggGTTCTTACCTTTCTTTTCCAATGTTACT	3, S5a-c	Primer F for pre-amplification step using RT-RPA - S ADESSO-Alpha, -Beta, -Omicron
S A67, HV69-70, D80 RT-	AAATAAACACCATCATTAAATGGTAGGAC	3, S5a-c	Primer R (1) for pre-amplification step using RT-RPA - S

RPA rev. primer 1			ADESSO-Alpha, -Beta, -Omicron
S A67, HV69-70, D80 RT-RPA rev. primer 2	GTAGGACAGGGTTATCAAACCTCTTAGTA	S5a	Primer R (2) for pre-amplification step using RT-RPA - S ADESSO-Alpha, -Beta, -Omicron
S T478 RT-RPA forward primer	gaaattaatacgactcactatagggGAAGTCTAATCTCAAACCTTTTGAGAGAGA	3, S5a-c	Primer F for pre-amplification step using RT-RPA - S ADESSO-Delta
S T478 RT-RPA rev. primer 1	GGTTGGAAACCATATGATTGTAAAGGAAAG	S5a	Primer R (1) for pre-amplification step using RT-RPA - S ADESSO-Delta
S T478 RT-RPA rev. primer 2	ATGATTGTAAAGGAAAGTAACAATTAAAACCTTCAA	3, S5a-c	Primer R (2) for pre-amplification step using RT-RPA - S ADESSO-Delta
**crRNAs**			
S crRNA (IVT template)	CTTCTTCAGGTTGGACAGCTGGTGCTGC**GTTTTAGTCCCCTTCGTTTTTGGGGTAGTCTAAATC**ccctatagtgagtcgtattaatttc	1-3, S1-S5	LwaCas13a crRNA IVT template - crRNA S gene
S crRNA	**GAUUUAGACUACCCCAAAAACGAAGGGGACUAAAAC**GCAGCACCAGCUGUCCAACCUGAAGAAG	1-3, S1-S5	crRNA resulting from IVT
Orf1a crRNA 1 (IVT template)	ATAGTTTAAAAATTACAGAAGAGGTTGG**GTTTTAGTCCCCTTCGTTTTTGGGGTAGTCTAAATC**ccctatagtgagtcgtattaatttc	S1c	LwaCas13a crRNA IVT template - crRNA orf1a gene
Orf1a crRNA	**GAUUUAGACUACCCCAAAAACGAAGGGGACUAAAAC**CCAACCUCUUCUGUAAUUUUUAAACUAU	S1c	crRNA resulting from IVT

S HV69-70 crRNA (IVT template)	TCCATGCTATACATGTCTCTGGGACCAA**GTTTTAGTCCCCTTCGTTTTTGGGGTAGTCTAAATC**ccctatagtgagtcgtattaatttc	S5a	LwaCas13a crRNA IVT template - crRNA S gene targeting region containing HV69-70
S HV69-70 crRNA	**GAUUUAGACUACCCCAAAAACGAAGGGGACUAAAAC**UUGGUCCCAGAGACAUGUAUAGCAUGGA	S5a	crRNA resulting from IVT
S T478 (IVT template)	ACTGAAATCTATCAGGCCGGTAGCACAC**GTTTTAGTCCCCTTCGTTTTTGGGGTAGTCTAAATC**ccctatagtgagtcgtattaatttc	S5a	LwaCas13a crRNA IVT template - crRNA S gene targeting region containing T478
S T478 crRNA	**GAUUUAGACUACCCCAAAAACGAAGGGGACUAAAAC**GUGUGCUACCGGCCUGAUAGAUUUCAGU	S5a	crRNA resulting from IVT
S crRNA ADESSO-Alpha (IVT template)	GGTTCCATGCTATCTCTGGGACCTAAGG**GTTTTAGTCCCCTTCGTTTTTGGGGTAGTCTAAATC**ccctatagtgagtcgtattaatttc	3, S5b,c	LwaCas13a crRNA IVT template - probe S gene specific for del69-70 for Alpha variant
S crRNA ADESSO-Alpha	**GAUUUAGACUACCCCAAAAACGAAGGGGACUAAAAC**CC**U**U**A**GGUCCCAGAGAUAGCAUGGAACC	3, S5b,c	crRNA resulting from IVT
S crRNA ADESSO-Beta (IVT template)	GGGAGCAATGGTACTAAGAGGTTAGCTA**GTTTTAGTCCCCTTCGTTTTTGGGGTAGTCTAAATC**ccctatagtgagtcgtattaatttc	3, S5b,c	LwaCas13a crRNA IVT template - probe S gene specific for D80A for Beta variant
S crRNA ADESSO-Beta	**GAUUUAGACUACCCCAAAAACGAAGGGGACUAAAAC**UA**G**C**U**AACCUCUUAGUACCAUUG**C**UCCC	3, S5b,c	crRNA resulting from IVT

S crRNA ADESSO-Delta (IVT template)	ACTGTAATCTATCAGGCCGGTAGGAAAC**GTTTTAGTCCCCTTCGTTTTTGGGGTAGTCTAAATC**ccctatagtgagtcgtattaatttc	3, S5b,c	LwaCas13a crRNA IVT template - probe S gene specific for T478K for Delta variant
S crRNA ADESSO-Delta	**GAUUUAGACUACCCCAAAAACGAAGGGGACUAAAAC**GU**U**U**C**CUACCGGCCUGAUAGAUU**A**CAGU	3, S5b,c	crRNA resulting from IVT
S crRNA ADESSO-Omicron (IVT template)	CCAAAGTTACTTGGTTCCATGTTTTGTC**GTTTTAGTCCCCTTCGTTTTTGGGGTAGTCTAAATC**ccctatagtgagtcgtattaatttc	3, S5b,c	LwaCas13a crRNA IVT template - probe S gene specific for A67V+del69-70 for Omicron variant
S crRNA ADESSO-Omicron	**GAUUUAGACUACCCCAAAAACGAAGGGGACUAAAAC**GA**C**A**A**A**A**CAUGGAACCAAGUAAC**U**UUGG	3, S5b,c	crRNA resulting from IVT
**RNA reporters**			
Lateral Flow RNA reporter	56-FAM/rUrUrUrUrUrU/3Bio	1-3, S1-S5	
Fluorescence RNA reporter	5TEX615/T*A*rArUG*C*/3IAbRQSp	1, S3	

Supplementary Table 6: Oligonucleotides used in this study. T7-3G sequence (or its complementary in the IVT templates oligos) is written in lowercase letters. Direct repeat sequences (or their complementary in the IVT templates oligos) of the crRNAs are highlighted in bold. The bases pairing with the specific mutation to be detected are highlighted in bold-red, and the synthetic mismatches[3] are highlighted in bold-blue.

Reagent	Reaction	Details
plasmid # 90097	Cas13 synthesis	Addgene
Rosetta cells	Cas13 synthesis	See Kellner et al 2019
Terrific Broth	Cas13 synthesis	See Kellner et al 2019
Ampicillin	Cas13 synthesis	See Kellner et al 2019
IPTG	Cas13 synthesis	See Kellner et al 2019
Lysis Buffer	Cas13 synthesis	See Kellner et al 2019
Complete Ultra EDTA-free tablets	Cas13 synthesis	See Kellner et al 2019
Lysozyme	Cas13 synthesis	See Kellner et al 2019
Benzoase	Cas13 synthesis	See Kellner et al 2019
Tris	Cas13 synthesis	See Kellner et al 2019
NaCl	Cas13 synthesis	See Kellner et al 2019
DTT	Cas13 synthesis	See Kellner et al 2019
SUMO protease	Cas13 synthesis	See Kellner et al 2019
HEPES	Cas13 synthesis	See Kellner et al 2019
MgCl2	Cas13 synthesis	See Kellner et al 2019
gel filtration buffer	Cas13 synthesis	See Kellner et al 2019
synthetic SARS-CoV-2 RNA	RT-RPA (ADESSO)	Twist Biosciences MT007544.1 or MN908947.3
SARS-CoV-2 RNA	RT-RPA (ADESSO)	Prof. Bartenschlager (DKFZ, Heidelberg
OneStep RT-PCRkit	RT-PCR	Qiagen, #210212
PCR clean-up kit	RT-PCR?	Macherey-Nagel, #740609.250
HiScribe T7 Quick High Yield RNA Synthesis Kit	IVT	NEB, #E2050S

Monarch RNA Cleanup Kit	IVT	NEB, #T2050
Qubit RNA broad range (BR) kit	IVT	ThermoFisher Scientific, #Q10211
Sterile 0.9% saline	gargle sample collection	Fa. Fresenius Kabi, Bad Homburg, Germany
flocked swabs	swab sample collection	(Improswab, Fa. Improve Medical Instruments, Guanzhou/China
cobas 6800 system kit	RT-qPCR (Mannheim)	Roche, Penzberg, Germany
QIAamp® Viral RNA Mini kit	RNA extraction	Qiagen, #52904
Nucleic Acid isolation Kit I	RNA extraction	Roche
CDC taqman RT-qPCR	DKFZ RT-qPCR	
Superscript III One-Step RT-PCR kit with Platinum Taq Polymerase	DKFZ RT-qPCR	
Sarbeco E-Gen-Kit	RT-qPCR (Mannheim)	Fa. Tib Molbiol, Berlin, Germany
QuickExtract DNA Extraction solution	sample lysis (direct ADESSO)	Lucigen, #QE09050
Rnase Inhibitor, Murine	sample lysis, RT-RPA, fluorescence Cas13 reaction (ADESSO and direct ADESSO)	NEB, #M0314
Agencourt RNAClean XP Kit	IVT	Beckman Coulter, #A63987
Mini-PROTEAN TBE-Ureaprecast gel 10%	IVT	Bio-Rad #4566033
TwistAmp Basic	RT-RPA (ADESSO)	TwistDx, #TABAS03KIT
M-MuLV Reverse Transcriptase	RT-RPA (ADESSO)	NEB, #M0253
*Lwa*Cas13a protein	Cas13 reaction (ADESSO)	N/A

nuclease-free water	Cas13 reaction (ADESSO)	ThermoFisher Scientific, #AM9937
Trizma® hydrochloride solution pH 7.4 1M	Storage and Cleavage Buffer for Cas13 reaction (ADESSO)	Sigma-Aldrich, #T2194
NaCl 5M	Storage Buffer for Cas13 reaction (ADESSO)	Sigma-Aldrich, cat. no. S7653
Glycerol	Storage Buffer for Cas13 reaction (ADESSO)	Carl Roth, #6967.1
SUPERase-In RNase inhibitor	Cas13 reaction (ADESSO)	ThermoFisher Scientific, #AM2694
rNTP solution mix	Cas13 reaction (ADESSO)	NEB, #N0466
NxGen T7 RNA Polymerase	Cas13 reaction (ADESSO)	Lucigen, #30223-2
$MgCl_2$	Cas13 reaction (ADESSO)	Invitrogen, #AM9530G
HybriDetect — Universal Lateral Flow Assay Kit	lateral flow assay (ADESSO)	Milenia BiotecGmbH, Gießen, #MGHD 1
RNaseAlert Lab Test Kit v2	RNase activity detection assay	Thermo Fisher Scientific, #4479768
RIDA QUICK SARS-CoV-2 Antigen test	Antigen test	R-Biopharm AG, #N6803

Supplementary Table 7: Reagents and materials used in this study.

Supplementary Methods:
Below, we provide specific details for each experiment shown in this study.

**Fig. S1c (target: S, Orf1a) and S1d (target: S)**	
**RT-RPA (1x)**	**Volume (µl)**
Rehydration Buffer (RB)	5.9
F primer (10µM)	0.5
R primer (10µM)	0.5
M-MuLV RT (5 U/µl)	1.6
Sample	1.0
MgOAc	0.5
**Total volume**	**10**
Incubation at 42°C for 25 min	
**Cas13 detection**	**Volume (µl)**
Cleavage buffer (400mM Tris pH 7.4)	2
Nuclease-free water	9.6
LwaCas13a protein in SB (63.3µg/ml)	2
crRNA (10ng/µl)	1
Lateral-Flow-Reporter (20µM)	1
SUPERase-In Rnase Inhibitor	1
Lucigen T7 Polymerase	0.6
rNTP solution mix (25mM each)	0.8
$MgCl_2$ (120mM)	1
RPA reaction step	1
**Total volume**	**20**
Incubation at 37°C for 30 minutes	

Fig. 1b (target: S)				
**RT-RPA (1x)**	**Volume (µl)**			
	**Protocol 1**	**Protocol 2**	**Protocol 3**	**Protocol 4**
Rehydration Buffer (RB)	5.9	5.9	5.9	5.9
F primer (1, 2, 3: 20µM; 4: 50µM)	0.25	0.25	0.25	0.1
R primer (1, 2, 3: 20µM; 4: 50µM)	0.25	0.25	0.25	0.1
M-MuLV (200U/µl - M0253)	1.8	1	0.3	0.6
RNase Inhibitor, Murine (40U/µl - M0314)	0.3	0.3	0.3	0.3
Sample	1	1.8	2.5	2.5
MgOAc	0.5	0.5	0.5	0.5
**Total volume**	**10**	**10**	**10**	**10**
Incubation at 42°C for 25 min				
**Cas13 detection**	**Volume (µl)**			
Cleavage buffer (400mM Tris pH 7.4)	2			
Nuclease-free water	9.6			
LwaCas13a protein in SB (63.3µg/ml)	2			
crRNA (10ng/µl)	1			
Lateral-Flow-Reporter (20µM)	1			
SUPERase-In Rnase Inhibitor	1			
Lucigen T7 Polymerase	0.6			
rNTP solution mix (25mM each)	0.8			
$MgCl_2$ (120mM)	1			
RPA reaction step	1			
**Total volume**	**20**			

Incubation at 37°C for 30 minutes

Fig. S3a,b (target: S)					
**RT-RPA**	**Volume (µl)**				
	**1x**	**2x**	**3x**	**4x**	**5x**
Rehydration Buffer (RB)	5.9	11.8	17.7	23.6	29.5
F primer (20µM)	0.25	0.5	0.75	1	1.25
R primer (20µM)	0.25	0.5	0.75	1	1.25
M-MuLV RT (200U/µl - M0253)	0.30	0.6	0.9	1.2	1.5
Rnase Inhibitor Murine (40U/µl - M0314)	0.3	0.6	0.9	1.2	1.5
Sample	2.5	5	7.5	10	12.5
MgOAc	0.5	1	1.5	2	2.5
**Total volume**	**10**	**20**	**30**	**40**	**50**
Incubation at 42°C for 25 min					
**Cas13 detection**	**Volume (µl)**				
Cleavage buffer (400mM Tris pH 7.4)	2				
Nuclease-free water	9.6				
LwaCas13a protein in SB (63.3µg/ml)	2				
crRNA (10ng/µl)	1				
Lateral-Flow-Reporter (20µM)	1				
SUPERase-In Rnase Inhibitor	1				
Lucigen T7 Polymerase	0.6				
rNTP solution mix (25mM each)	0.8				
$MgCl_2$ (120mM)	1				
RPA reaction step	1				

**Total volume**	**20**
Incubation at 37°C for 30 minutes	

**Fig. S3c,d (target: S)**			
**Cas13 detection (fluorescence readout)**	**Volume (µl)**		
	**Cas13 45nM + crRNA 22.5nM**	**Cas13 45nM + crRNA 45nM**	**Cas13 90nM + crRNA 90nM**
Nuclease-free water	8.6	8.6	8.6
Cleavage buffer (400mM Tris pH 7.4)	2	2	2
LwaCas13a protein (63.3µg/ml; 126.6µg/ml)	2	2	2
crRNA (10ng/µl; 20ng/µl; 40ng/µl)	1	1	1
LwaCas13a specific fluo reporter (2µM; 4µM)	1	1	1
Murine Rnase Inhibitor (40 U/µl)	1	1	1
rNTP solution mix (25mM each)	0.8	0.8	0.8
Lucigen T7 RNA Polymerase (50 U/µl)	0.6	0.6	0.6
$MgCl_2$ (120mM)	1	1	1
Total divided by number of samples	18.0	18.0	18.0
RPA reaction	2.0	2.0	2.0
**Total volume**	**20.0**	**20.0**	**20.0**
Incubation at 37°C for 3 hours, measurement every 5 minutes			

Fig. S3e,f (target: S)	
**RT-RPA (2x)**	**Volume (µl)**
Rehydration Buffer (RB)	11.8
F primer (20µM)	0.5
R primer (20µM)	0.5
M-MuLV RT (200U/µl - M0253)	0.3
Rnase Inhibitor Murine (40U/µl - M0314)	0.3
Sample	5.6
MgOAc	1
**Total volume**	**20**
Incubation at 42°C for 25 min	
**Cas13 detection (half volume)**	**Volume (µl)**
Nuclease-free water	4.3
Cleavage buffer (400mM Tris pH 7.4)	1
LwaCas13a protein in 74µl SB (126.6µg/ml)	1
crRNA (40ng/µl)	0.5
Lateral-Flow-Reporter (20µM)	0.5
SUPERase-In Rnase Inhibitor (20U/µl)	0.5
rNTP solution mix (25mM each)	0.4
Lucigen T7 RNA Polymerase	0.3
$MgCl_2$ (120mM)	0.5
RPA reaction step	1
**Total volume**	**10**
Incubation at 37°C for 5-10-15-20-25-30 minutes (negative control 30 min)	

Fig. 1d-e, 2, 3b, S4, S5a (target: S, S HV69-70, S T478)	
**RT-RPA 2x**	**Volume (µl)**
Rehydration Buffer (RB)	11.8
F primer (20µM)	0.5
R primer (20µM)	0.5
M-MuLV RT (200U/µl - M0253)	0.3
Rnase Inhibitor Murine (40U/µl - M0314)	0.3
Sample	5.6
MgOAc	1
**Total volume**	**20**
Incubation at 42℃ for 45 min	
**Cas13 detection (half volume)**	**Volume (µl)**
Nuclease-free water	4.3
Cleavage buffer (400mM Tris pH 7.4)	1
LwaCas13a protein in 74µl SB (126.6µg/ml)	1
crRNA (40ng/µl)	0.5
Lateral-Flow-Reporter (20µM)	0.5
SUPERase-In Rnase Inhibitor	0.5
rNTP solution mix (25mM each)	0.4
Lucigen T7 RNA Polymerase	0.3
$MgCl_2$ (120mM)	0.5
RPA reaction step	1
**Total volume**	**10.0**
Incubation at 37°C for 10 minutes	

Fig. 3b, S5b-e (target: S HV69-70del, S D80A, S A67V+HV69-70del, S T478K)	
**RT-RPA 2x**	**Volume (µl)**
Rehydration Buffer (RB)	11.8
F primer (20µM)	0.5
R primer (20µM)	0.5
M-MuLV RT (200U/µl - M0253)	0.3
Rnase Inhibitor Murine (40U/µl - M0314)	0.3
Sample	5.6
MgOAc	1
**Total volume**	**20**
Incubation at 42℃ for 45 min	
**Cas13 detection (half volume)**	**Volume (µl)**
Nuclease-free water	4.3
Cleavage buffer (400mM Tris pH 7.4)	1
LwaCas13a protein in 74µl SB (126.6µg/ml)	1
crRNA (40ng/µl)	0.5
Lateral-Flow-Reporter (20µM)	0.5
SUPERase-In Rnase Inhibitor	0.5
rNTP solution mix (25mM each)	0.4
Lucigen T7 RNA Polymerase	0.3
$MgCl_2$ (120mM)	0.5
RPA reaction step	1
**Total volume**	**10.0**
Incubation at 37°C for 20 minutes	

Supplementary References

1. Kellner, M. J., Koob, J. G., Gootenberg, J. S., Abudayyeh, O. O. & Zhang, F. SHERLOCK: nucleic acid detection with CRISPR nucleases. *Nat. Protoc.* **14**, 2986–3012 (2019).
2. Zhang, F., Abudayyeh, O. O. & Gootenberg, J. S. A protocol for detection of COVID-19 using CRISPR diagnostics. (2020).
3. Gootenberg, J. S. *et al.* Nucleic acid detection with CRISPR-Cas13a/C2c2. *Science* **356**, 438–442 (2017).
