# Supplementary material for: Rapid, adaptable and sensitive Cas13-based COVID-19 diagnostics using ADESSO
Source: Nat Commun. 2022 Jun 8;13:3308. doi: 10.1038/s41467-022-30862-y (PMC9176161; doi:10.1038/s41467-022-30862-y)
Supplement: Supplementary file 3 — Description of Additional Supplementary Files [file 41467_2022_30862_MOESM3_ESM.pdf]

Description of Additional Supplementary Files

File Name: Supplementary Data 1
Description: This file contains all the sequencing information about variant samples

File Name: Supplementary Data 2
Description: This file contains all the SNVs in S shown in Supplementary Fig. 5e.

File Name: Supplementary Data 3
Description: This file contains the frequencies of SNVs listed in Supplementary File 2 and shown in Supplementary Fig. 5e.

File Name: Supplementary Data 4
Description: This file contains the deletions in S and their frequencies shown in Supplementary Fig. 5e.
