# Supplementary material for: Rapid, adaptable and sensitive Cas13-based COVID-19 diagnostics using ADESSO
Source: Nat Commun. 2022 Jun 8;13:3308. doi: 10.1038/s41467-022-30862-y (PMC9176161; doi:10.1038/s41467-022-30862-y)
Supplement: Supplementary file 8 — Reporting summary [file 41467_2022_30862_MOESM8_ESM.pdf]

nature portfolio

Corresponding author(s): Riccardo Pecori, Fotini Nina Papavasiliou, Thomas Miethke

Last updated by author(s): Jun 3, 2022

Reporting Summary

Nature Portfolio wishes to improve the reproducibility of the work that we publish. This form provides structure for consistency and transparency in reporting. For further information on Nature Portfolio policies, see our Editorial Policies and the Editorial Policy Checklist.

Statistics

For all statistical analyses, confirm that the following items are present in the figure legend, table legend, main text, or Methods section.

n/a	Confirmed	
☐	☒	The exact sample size (*n*) for each experimental group/condition, given as a discrete number and unit of measurement
☐	☒	A statement on whether measurements were taken from distinct samples or whether the same sample was measured repeatedly
☐	☒	The statistical test(s) used AND whether they are one- or two-sided<br>*Only common tests should be described solely by name; describe more complex techniques in the Methods section.*
☒	☐	A description of all covariates tested
☒	☐	A description of any assumptions or corrections, such as tests of normality and adjustment for multiple comparisons
☐	☒	A full description of the statistical parameters including central tendency (e.g. means) or other basic estimates (e.g. regression coefficient) AND variation (e.g. standard deviation) or associated estimates of uncertainty (e.g. confidence intervals)
☐	☒	For null hypothesis testing, the test statistic (e.g. *F*, *t*, *r*) with confidence intervals, effect sizes, degrees of freedom and *P* value noted<br>*Give P values as exact values whenever suitable.*
☒	☐	For Bayesian analysis, information on the choice of priors and Markov chain Monte Carlo settings
☒	☐	For hierarchical and complex designs, identification of the appropriate level for tests and full reporting of outcomes
☐	☒	Estimates of effect sizes (e.g. Cohen's *d*, Pearson's *r*), indicating how they were calculated

Our web collection on statistics for biologists contains articles on many of the points above.

Software and code

Policy information about availability of computer code

Data collection	A fluorescence plate reader were used to record fluorescence signals from ADESSO detection of viral RNA. Real-time PCR systems were used to record cycle threshold values for RNA standards and clinical RNA samples. When lateral flow readout was used for ADESSO testing the image was photographed with a smartphone camera and analysed by ImageJ.
Data analysis	ImageJ (v1.53a) was used to measure the intensity of the bands when lateral flow readout was used. This is also mentioned in the Methods section. Data panels and statistical analysis were generated and performed using Prism 8 (GraphPad).

For manuscripts utilizing custom algorithms or software that are central to the research but not yet described in published literature, software must be made available to editors and reviewers. We strongly encourage code deposition in a community repository (e.g. GitHub). See the Nature Portfolio guidelines for submitting code & software for further information.

Data

Policy information about availability of data

All manuscripts must include a data availability statement. This statement should provide the following information, where applicable:

- Accession codes, unique identifiers, or web links for publicly available datasets
- A description of any restrictions on data availability
- For clinical datasets or third party data, please ensure that the statement adheres to our policy

All the information of the clinical samples used in this work are available in Supplementary Table 1-4.
All the SARS-CoV-2 genomes sequences analyzed here were downloaded from GISAID.

DATA AVAILABILITY

Primer and crRNA design. Primers for RT-RPA and crRNAs for Cas13 detection were designed following the guidelines published for the SHERLOCK method (ref 40) using NCBI Primer-BLAST (ref 64), Primer3Plus (ref 65) or ADAPT (ref 66). Specific information about each primer and crRNA is provided in Supplementary Table 6.

Human clinical specimen. Information regarding all the samples used in this study is available in Supplementary Tables 1-4. Sequencing information reporting mutations within the S gene for clinical samples carrying SARS-CoV-2 variants is available in Supplementary Data 1.

Protocols. The RT-RPA and Cas13 reaction protocols used for each experiment are provided in Supplementary Methods with reference to the corresponding figures. The exact volumes are given for one single reaction.

Reagents and materials. Detailed information about reagents and material used in this study is provided in Supplementary Table 7.

Bioinformatic analysis database and output files. All the SARS-CoV-2 genomes sequences analyzed here were downloaded from GISAID (ref 67). All the output files are available (Supplementary Data 2-4).

Field-specific reporting

Please select the one below that is the best fit for your research. If you are not sure, read the appropriate sections before making your selection.

- [x] Life sciences
- [ ] Behavioural & social sciences
- [ ] Ecological, evolutionary & environmental sciences

For a reference copy of the document with all sections, see nature.com/documents/nr-reporting-summary-flat.pdf

Life sciences study design

All studies must disclose on these points even when the disclosure is negative.

Sample size	195 samples (95 positive and 100 negative) were used for the comparison shown in Figure 2. These 195 samples were tested in parallel with 4 different methods, for a total of 780 samples for each sampling method. Additionally, 30 more samples were analyzed in Figure S1, and another 20 in Figure 3. No selection of any kind was performed based on Ct values. These parameters allowed us to avoid bias due to sample size or viral load distribution based on reference n°43.
Data exclusions	Originally 100 positive samples were included in the comparison based on COBAS RT-qPCR (see Figure 2a). However, 5 of them resulted negative for any detection method used during phase 3. Therefore, we couldn't exclude that they were false positive samples and therefore we decided to exclude them from the study.
Replication	All the experiments using viral synthetic genome were repeated at least three times. Experiments with samples testing were only performed once in the attempt to reproduce as close as possible a "real life" scenario. All attempts at replication were successful.
Randomization	All the clinical samples used in this study were randomly collected from ambulatory patients presenting minimal to mild symptoms or sent by the German Health Department after having contact with a SARS-CoV-2 positive person, without any kind of selection.
Blinding	ADESSO testing, RT-qPCR and antigen tests were performed blindly, without previous knowledge about the status (positivity or negativity) of the samples.

Reporting for specific materials, systems and methods

We require information from authors about some types of materials, experimental systems and methods used in many studies. Here, indicate whether each material, system or method listed is relevant to your study. If you are not sure if a list item applies to your research, read the appropriate section before selecting a response.

Materials & experimental systems

n/a	Involved in the study	
[ ]	[x]	Antibodies
[x]	[ ]	Eukaryotic cell lines
[x]	[ ]	Palaeontology and archaeology
[x]	[ ]	Animals and other organisms
[ ]	[x]	Human research participants
[x]	[ ]	Clinical data
[x]	[ ]	Dual use research of concern

Methods

n/a	Involved in the study	
[x]	[ ]	ChIP-seq
[x]	[ ]	Flow cytometry
[x]	[ ]	MRI-based neuroimaging

Antibodies

Antibodies used	The antibodies were provided with the Milenia Biotec kit for the lateral-flow read out. The kit uses a polyclonal (rabbit) anti-FITC antibody labelled with gold particles.

Validation	Validation was performed by the provider (Milenia Biotec).

Human research participants

Policy information about studies involving human research participants

Population characteristics	The only information available for the clinical samples used in this study is the Ct value. And our randomly selected cohort of positive individuals covers the full distribution of viral titers between Ct 17 and Ct 38.
Recruitment	Clinical specimens were randomly collected from ambulatory patients presenting minimal to mild symptoms or sent by the German Health Department after having contact with a SARS-CoV-2 positive person. No personal identifiable information were collected.
Ethics oversight	Ethics Committee II of the University of Heidelberg.

Note that full information on the approval of the study protocol must also be provided in the manuscript.
